# Corrosion and Residual Strength Analysis of High Pressure Die Casting AM Series Mg Alloys

**DOI:** 10.3390/ma12162624

**Published:** 2019-08-17

**Authors:** Tianxu Zheng, Yaobo Hu, Wanqiu Meng, Aitao Tang, Fusheng Pan

**Affiliations:** 1 State Key Laboratory of Mechanical Transmission, College of Materials Science and Engineering, Chongqing University, Chongqing 400044, China; 2 National Engineering Research Center for Magnesium Alloys, Chongqing 400044, China

**Keywords:** magnesium alloys, corrosion residual strength, AM series

## Abstract

Higher pressure die casting (HPDC) AM series (Mg-Al-Mn) Mg alloys have wide application potential in the automobile industry. To promote its application, systematic investigation on the corrosion performance and corrosion residual strength of HPDC AM50+1Ce and AM60 was carried out. The corrosion of HPDC AM50+1Ce was more uniform, while the pitting corrosion of AM60 was more severe, and the mechanical properties of HPDC AM60 was more sensitive to corrosion. The residual strength of AM50+1Ce and AM60 after corrosion of 648 h was 199 MPa and 183 MPa, respectively. The findings can contribute to a better understanding of the corrosion and residual strength of HPDC AM series Mg alloys.

## 1. Introduction

Fuel consumption of automobiles is huge, therefore improving the fuel economy of the automobile and reducing emissions are inevitable trends [1]. One way to improve vehicle fuel efficiency is to utilize lighter materials in the transportation industry [2,3]. As the lightest metal structural material, Mg alloys attract much attention for its broad application prospects in lightweight automobiles [4,5,6,7].

In recent years, there has been extensive research on Mg alloys [8,9,10,11,12,13,14,15], especially in wrought Mg alloys [16,17,18,19,20,21]. However, almost no Mg alloys are utilized in the automobile industry other than high pressure die casting (HPDC) Mg alloys. The main application of HPDC Mg alloys includes door frames, dashboards, wheel hubs, steering wheels and decorative components [22]. AM series Mg alloys have good comprehensive properties, therefore much effort has been made to expand their application range, and with the boost of new energy vehicles and lightweight automobile wave, applications of AM series (Mg-Al-Mn) Mg alloys for automotive applications will continue to increase. One of the fundamental challenges preventing widespread use of HPDC AM series Mg alloys, however, is their poor corrosion resistance, especially in the humid air environment and in the environment containing anions such as Cl^−^, Br^−^, ClO_4_^−^ and SO_4_^2−^ [23,24]. Therefore, research work on the corrosion mechanism of HPDC AM series Mg alloys is urgently needed, as well as the effect of corrosion on the mechanical properties.

In addition, many studies have pointed out that the addition of rare earth elements in Mg alloy can improve the corrosion performance of magnesium alloy and improve the mechanical properties as well. Cerium is one of the most commonly used rare earth elements. The addition of rare earth Ce can improve the corrosion resistance of AZ91 [25,26,27,28], AZ61 [29], AM60 [30,31,32] and WE43 [33] and other magnesium alloys, mainly due to purifying effects. Ce has a very low solid solubility in the α-Mg matrix, Al can react with Ce to form Al–Ce compounds, which is less cathodic than M_17_Al_12_, thus reduces the micro-galvanic couple, thereby the corrosion resistance of magnesium alloy enhances.

In the present study, the corrosion property of HPDC AM60 and AM50+1Ce was systematically investigated through hydrogen evolution, weight loss and electrochemical tests. The corrosion residual strength and corrosion mechanism were also included. Exploring of this work may provide ideas for corrosion protection of AM series Mg alloys and lay a foundation for further promotion of the industrial application of HPDC AM series Mg alloys.

## 2. Materials and Methods 

### 2.1. Hydrogen Evolution

**Figure 1:**
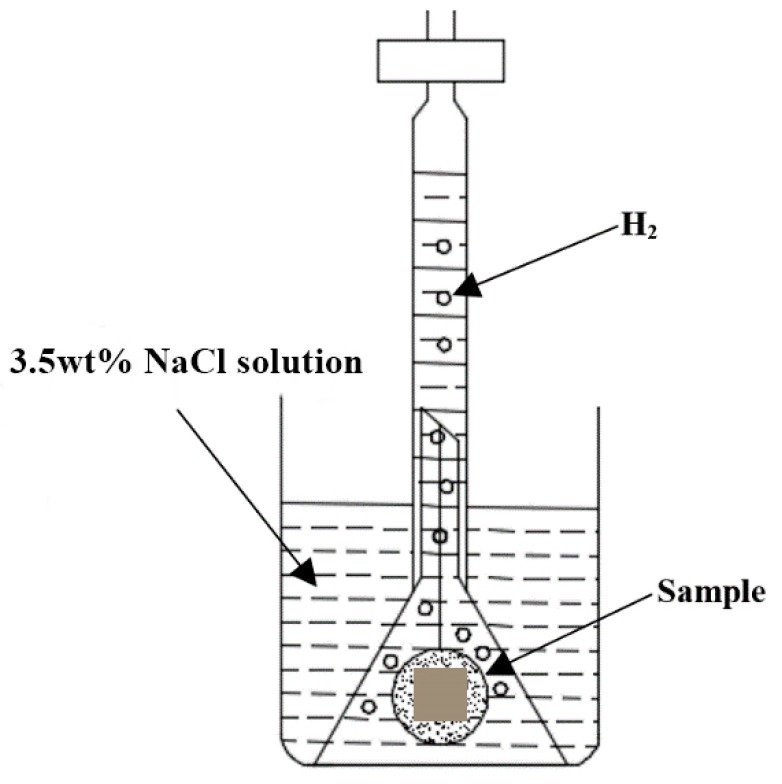
Schematic illustration of hydrogen evolution apparatus.

The HPDC AM60 and AM50+1Ce were processed into square samples with a dimension of 10 mm × 10 mm × 5 mm. Firstly, the surface of the sample was ground with SiC sandpaper (up to 200 grit), and cleaned with ethanol in an ultrasonic cleaner for 10 min. Then the samples were sealed with resin to expose only the working surface of 10 mm × 10 mm, and the working surface of the sample was sequentially polished with SiC sandpaper (up to 1000 grit). After being cleaned with ethanol in the ultrasonic cleaner again, the samples were taken out and dried with cold air. The schematic diagram of the hydrogen evolution test is illustrated in Figure 1. The samples were tied with a thin wire, then put into a beaker containing 200 mL 3.5 wt% NaCl solution, an inverted burette with a funnel were used to collect and measure the evolved hydrogen, and the data was collected over 132 h. In addition, much attention was paid to make sure the working surface of the sample was vertical to the bottom of the beaker during the experiment.

### 2.2. Weight Loss Experiment

**Figure 2:**
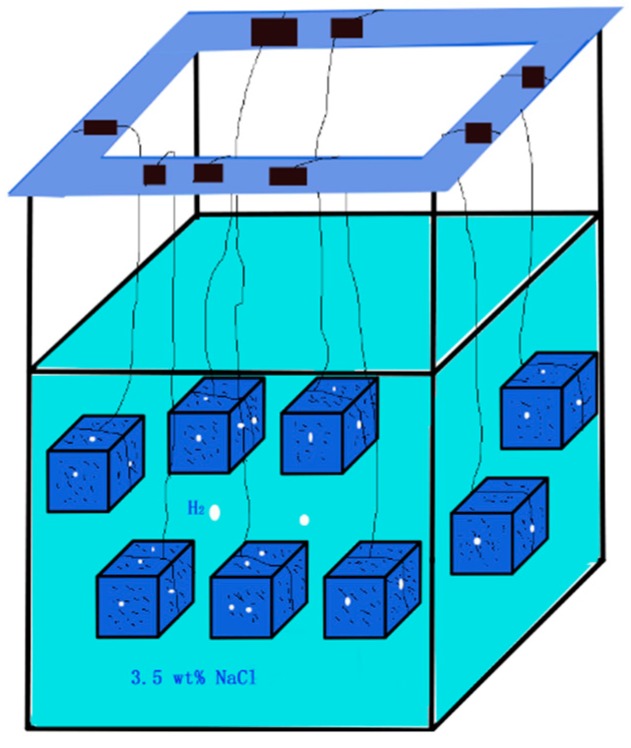
Schematic diagram and actual picture of the weight loss experiment.

Samples for weight loss were cut from the samples provided and machined into a cuboid with a dimension of 10 mm × 10 mm × 5 mm. Before the immersion experiment, the six faces of the weight loss sample were first grounded with SiC sandpaper (up to 1000 grit). Then cleaned with anhydrous alcohol in an ultrasonic cleaner for 5 min, and rinsed with anhydrous alcohol again, then dried with cold air. The actual dimension of the weight loss samples was measured with a Vernier caliper, and the actual surface area *S*_0_ of each sample was calculated, then the initial weight was measured and recorded as *m*_0_. Each weight loss sample was labeled and sealed. The weight loss test was performed at time junctures of 24 h (1 day), 72 h (3 days), 168 h (7 days), 264 h (11 days), 456 h (19 days) and 648 h (27 days). As presented in Figure 2, the samples were tied with a thin wire and immersed in a 3.5 wt% NaCl solution during the experiment. After the immersion test, silver nitrate combining with chromic acid solution was used to clean the corrosion product, then the samples were rinsed with anhydrous alcohol and dried with cold wind, finally the weight after corrosion was measured and recorded as *m*_t_.

The corrosion rate was calculated according to the Equation (1), The average corrosion rates could also be calculated using the Equation (2):

(1) $$v_{t}=\frac{m_{0}-m_{t}}{S_{0}t}$$

(2) $$R_{\mathrm{corr}}=\frac{365\times 24\times 10^{-2}(m_{0}-m_{t})}{\rho S_{0}t}$$

where *v*_t_ represents the weight loss corrosion rate (mg·cm^−2^·h^−1^), *m*_0_ represents the initial weight (mg), *m*_t_ represents the weight after corrosion (mg), *S*_0_ is the actual surface area of the samples (cm^2^), *t* is the corrosion time (h), *R*_corr_ is the annual average corrosion rate (mm·y^−1^), *ρ* is the density of the alloy (g·cm^−3^), 1.80 g·cm^−3^ here.

### 2.3. Electrochemical Test

The electrochemical test was conducted using an electrochemical workstation (CHI660E, Shanghai, China and Zennium E, Kronach, Germany) within a three-electrode system, where the working electrode was a machined magnesium alloy sample (10 mm × 10 mm), the auxiliary electrode was a platinum electrode (15 mm × 15 mm), and the reference electrode was a supersaturated calomel electrode (SCE). The electrolyte was 3.5 wt% NaCl solution, which was prepared from analytically pure sodium chloride and distilled water, and the volume of the electrolyte used in each test was 100 mL. For open circuit potential time curves, the test was carried out in the CHI660E electrochemical workstation, the recording time was 1200 s. The polarization curves were tested on the CHI660E electrochemical workstation by potentiodynamic scanning. The working electrode was immersed in 3.5 wt% NaCl solution for about 5 min before the test to ensure that the open circuit potential stabilized. The scanning speed was 10 mV/s towards the positive direction. Electrochemical impedance spectroscopy (EIS) was tested on the Zennium E electrochemical workstation. The frequency range of the test was 10^−1^ to 10^5^ Hz, scanning from high frequency to range to low frequency range. The amplitude of the sinusoidal AC (Alternating Current) potential was 5 mV. Before the test, the working electrode was put into the solution for 5 min to stabilize the system. The equivalent circuit model was established by fitting the data using Zview software (Southern Pines, NC, USA, Version2.70)to reveal corrosion behavior of the HPDC AM60 and AM50+1Ce.

### 2.4. Residual Strength of Corroded Mg Alloy

**Figure 3:**
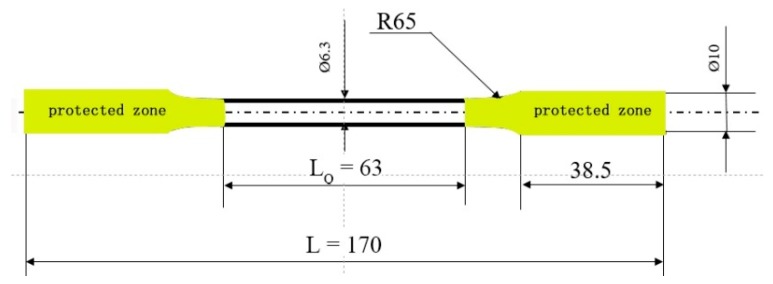
The tensile sample for the corrosion residual strength test.

The tensile samples as shown in Figure 3 were immersed in 3.5 wt% NaCl aqueous solution at room temperature for six sets of time, 24 h (1 day), 72 h (3 days), 168 h (7 days), 264 h (11 days), 456 h (19 days) and 648 h (27 days). Before soaking, the actual diameter of the two ends of the tensile specimen was measured with a Vernier caliper, at least 10 sets of data were collected for one sample, and then the ends of the sample sealed with insulating tape. After the sample was soaked and etched, the sample was taken out, washed with absolute ethanol, and placed in a pre-configured silver nitrate chromic acid solution to clean the corrosion product. After the corrosion product of the tensile sample was removed, it was taken out and rinsed with distilled water and ethanol, finally dried with cold air. Tensile properties were then tested on a universal material machine (GMT-5105, Shanghai, China) with a tensile rate of 3 mm/min. The test was repeated three times for each set of samples to ensure the accuracy of the experimental data. Since the surface of the tensile samples had been damaged after immersion, the actual cross-sectional area of the gauge length of the tensile specimen was difficult to measure, corrosion residual strength (*σ*_CRS_, MPa) was calculated using the Equation (3):

(3) $$\sigma_{\mathrm{CRS}}=A\frac{P}{S_{0}}$$

where *A* is the safety factor of product setting in practical application, which was set to 1 in the present study, *P* is the breaking load of the corroded sample (N), *S*_0_ is the original cross-sectional area of the sample (mm^2^). It is noteworthy that the corrosion residual strength is different from the residual strength of the corroded material. The decrease in the yield stress and ultimate tensile strength (UTS) can be related to the decrease in *S*_0_ due to corrosion, so we can obtain the information about the effect of corrosion on mechanical properties, which is important for the security of practical application.

### 2.5. Other Characterization

Fracture morphology and corrosion morphology were observed using scanning electron microscope (TESCANVEGA II LMU, Brno, Czech Republic). The actual compositions of alloys were detected by inductively coupled plasma optical emission spectrometry (ICP-OES, Optima 8000).

## 3. Results

### 3.1. Hydrogen Evolution, Weight Loss and Corrosion Residual Strength

**Figure 4:**
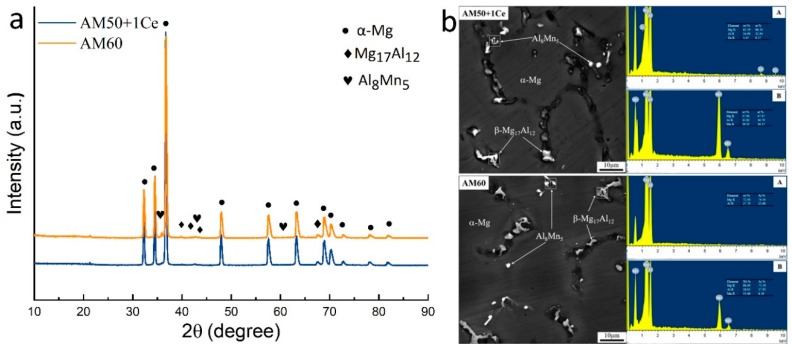
(**a**) XRD pattern and (**b**) SEM with EDS of higher pressure die casting (HPDC) AM50+1Ce and AM60 magnesium alloys.

**Figure 5:**
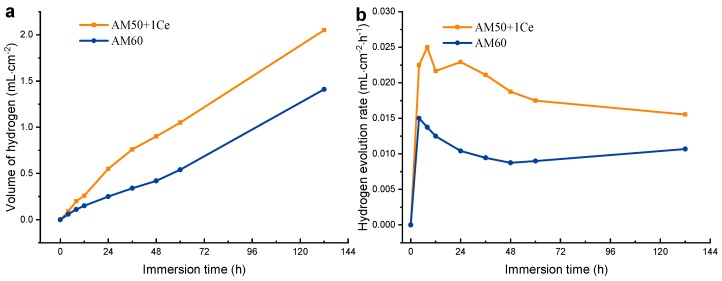
Volume of hydrogen evolved (**a**) and hydrogen rate (**b**) for HPDC AM series Mg alloys during 132 h immersion in 3.5 wt% NaCl solution.

Looking at the XRD pattern (Figure 4a), both alloys were composed of α-Mg, Mg_17_Al_12_ and Al_8_Mn_5_ phases, the result was further confirmed from Energy Dispersive Spectrometer (EDS) (Figure 4b). The actual composition of the tested alloy is presented in Table 1. Figure 5 shows the hydrogen evolution curves of HPDC AM50+1Ce and AM60 immersed in 3.5 wt% NaCl solution for 132 h. As immersion time extended, the amount of hydrogen evolution increased continuously. At 132 h, the hydrogen evolution of HPDC AM50+1Ce reached 2.05 mL/cm^2^, while the hydrogen evolution of AM60 was 1.41 mL/cm^2^, which was 0.849 mm·y^−1^ and 0.584 mm·y^−1^, respectively. The hydrogen evolution of HPDC AM50+1Ce was much larger than that of AM60, indicating that the self-corrosion rate of AM50+1Ce was greater than AM60. Looking at Figure 5b, the hydrogen evolution rate of HPDC AM50+1Ce was always greater than that of AM60 during the 132 h period of immersion test. In the initial stage, the corrosion rate of both alloys increased quickly, then the corrosion rate of both alloys dropped sharply during the 4–12 h period. When in contact with the corrosion media, a large amount of pitting occurred, which caused the increased corrosion rate. Then, the corrosion product and passivation film formed on the surface of samples, reducing the contact area between the substrate and corrosive media and obstructing the diffusion of Mg ions, which made the corrosion rate decrease. During the period of 12–24 h, the hydrogen evolution rate of the two went up again, as the formed passivation film was not dense enough, especially in the Cl^−^ containing solution. In the ionic solution, Cl^−^ ions could penetrate the oxidation film easily, destroying the protective film, and the hydrogen evolution rate became larger again. As the protective film thickened, the rate of hydrogen evolution slowed down again. During the 24–60 h period, the extension of pitting corrosion and the formation of the protective film reached a dynamic equilibrium, and the rate of hydrogen evolution did not change much. After 60 h, the hydrogen evolution rate of the two showed an increasing trend, which may have been the cause of the instability of the protective film, and may also be related to the complex corrosion behavior of magnesium alloys in aqueous Cl^−^ containing solutions. 

**Figure 6:**
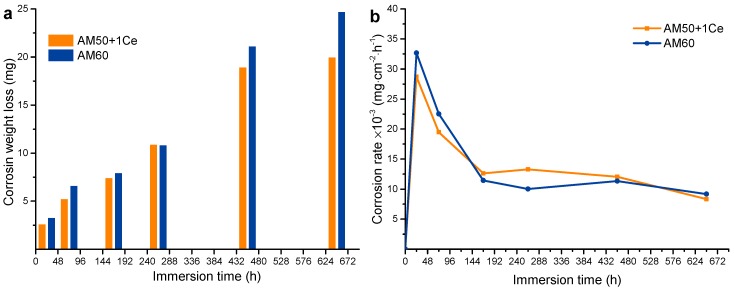
The weight loss (**a**) and corrosion weight rate (**b**) of HPDC AM50+1Ce and AM60 during immersion in 3.5 wt% NaCl solution for 648 h.

Figure 6 displays the weight loss and weight loss rate of HPDC AM50+1Ce and AM60 immersed in 3.5 wt% NaCl solution for 648 h. As the immersion time extended, the corrosion loss of the two samples both increased. It can be observed from Figure 6b that the weight loss rate of HPDC AM50+1Ce and AM60 rose sharply during the 0–24 h immersion stage, the weight loss rate of AM60 (9.162 × 10^−3^ mg·cm^−2^·h^−1^) was greater than AM50+1Ce (8.342 × 10^−3^ mg·cm^−2^·h^−1^), indicating that in the whole process of immersion for 648 h in 3.5 wt% NaCl solution, the average corrosion of HPDC AM60 was more serious. According to Equation (2), the annual average corrosion rates of HPDC AM50+1Ce and AM60 were calculated to be 0.406 mm·y^−1^ and 0.446 mm·y^−1^, respectively. The data from mass loss was close, and the data of mass loss was easily influenced in the corrosion product clean process, so a corrosion test through other methods was further conducted to compare the corrosion of these two alloys.

**Figure 7:**
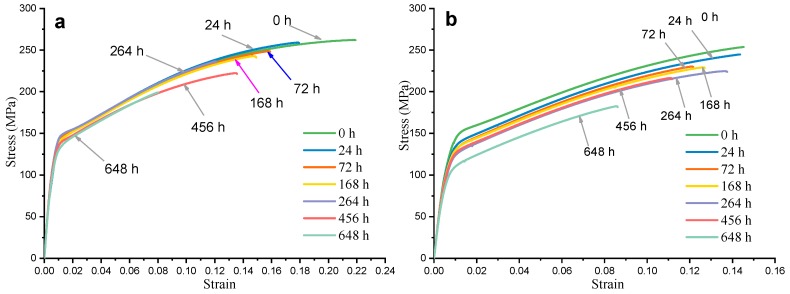
The engineering stress–strain curve of HPDC (**a**) AM50+1Ce and (**b**) AM60 magnesium alloys after corrosion test for different time immersing in 3.5 wt% NaCl solution.

**Figure 8:**
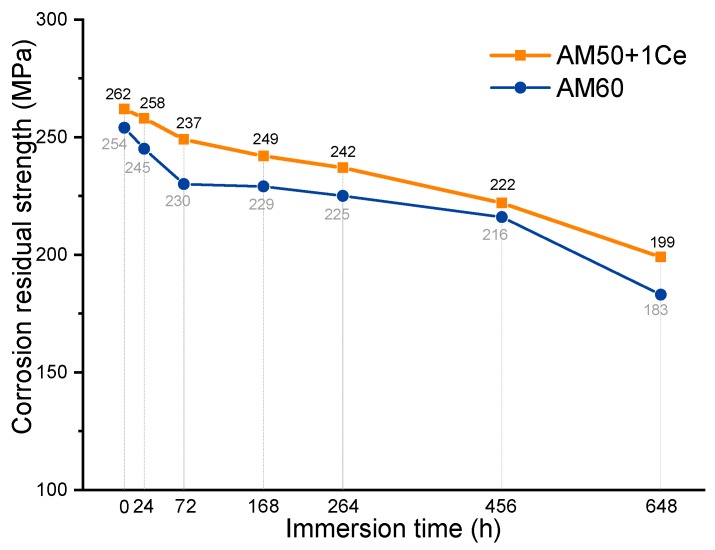
The corrosion residual strength comparison of HPDC AM50+1Ce and AM60 magnesium alloy for different corrosion times.

Figure 7 shows the engineering stress–strain curves obtained after corrosion of HPDC AM50+1Ce (a) and AM60 (b) in 3.5 wt% NaCl solution for 24 h, 72 h, 168 h, 264 h, 456 h and 648 h, respectively. The stress–strain curves at different corrosion time points all included the elastic deformation stage, the plastic deformation stage and the fracture. Corrosion did not change the deformation form of the magnesium alloy during the tensile process. It can be seen from Figure 7 that the yield strength, tensile strength and elongation of the two magnesium alloys tended to decrease with the extension of corrosion time. Figure 8 compares the corrosion residual strength of HPDC AM50+1Ce and AM60 immersed in 3.5 wt% NaCl solution for different times. During the immersion period of 0–72 h, the tensile strength of the two magnesium alloys declined significantly. In this process, the corrosion had just spread throughout the tensile specimen surface. During the subsequent 72–456 h period, the tensile strength of AM60 decreased little. In this process, a protective oxide film formed, but after immersion for 456 h, the oxide film was broken or damaged, which accelerated the corrosion of the magnesium alloy, resulting in a significant decrease in tensile strength. However, for HPDC AM50+1Ce magnesium alloy, there was no formation of a stable protective film after immersion for 72 h, the film layer fell off, the magnesium substrate was exposed, the corrosion aggravated, and the tensile strength showed a continuous decline. The corrosion residual strength of the two sets of samples after immersion for 648 h in 3.5 wt% NaCl solution was 199 MPa and 183 MPa, respectively, which were 63 MPa and 71 MPa lower than the immersion strength of 0 h, respectively. A simple comparison of the influence of corrosion on the strength shows that the greater the corrosion rate, the greater the rate of weight loss, and the more the cross-sectional area of the gauge section of the tensile specimen was reduced, the more the tensile strength decreased. The variation of the weight loss rate of HPDC AM50+1Ce and AM60 could reflect the corrosion rate of the alloy to some extent. However, many studies have shown that pitting corrosion is much more destructive to the substrate than uniform corrosion. The corrosion weight loss method is an average evaluation method for the corrosion rate, so it cannot accurately characterize the degree of local corrosion or pitting corrosion on the substrate, thus underestimating the harm of pitting. For Mg alloy under working load, pitting corrosion has significant influence on the mechanical properties of the alloy. Therefore, pitting depth is often used to evaluate the pitting corrosion. Therefore, experimental results of corrosion residual strength could only show that the pitting corrosion of AM60 was more serious, and its tensile strength was more likely to be influenced in the corrosion process in 3.5 wt% NaCl solution.

**Figure 9:**
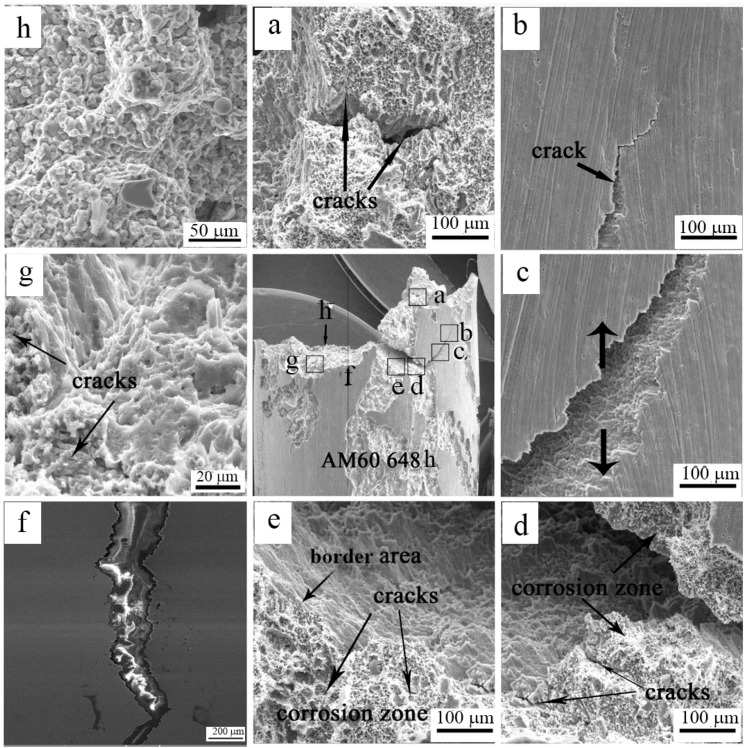
The tensile fracture surface of different zone (**a**–**h**) of HPDC AM60 magnesium alloy immersed in 3.5 wt% NaCl solution for 648 h.

To further investigate the effect of corrosion on the strength of HPDC AM60, after immersion in 3.5 wt% NaCl aqueous solution for 648 h, the fracture surface of AM60 was observed under scanning electron microscopy. As can be seen from (a), (d), (e) and (h) in Figure 9, a large number of micro cracks were formed at the interface between the corroded area and the intact matrix. Under the load condition, these micro cracks were preferentially formed in the pitting area and extended to the inside of the magnesium matrix. Fracture morphology near the corrosion zone (e) is different from the morphology in the interior (g) of the matrix. The fracture morphology of the transition zone has no dimples and no tearing edges, which was a brittle fracture. Due to the formation of a large number of micro cracks in the corrosion zone of the surface layer, the crack propagation grew and joined together, and it is easy to form a highly harmful tear pattern on the surface of the alloy, as shown in Figure 9b,c. Cracks expand rapidly, causing the material to break. Although the corrosion did not change the main plastic deformation mechanism and the final fracture mechanism inside the matrix, the appearance of a large number of cracks on the surface layer had a great influence on the fracture of the alloy.

### 3.2. Electrochemical Test

**Figure 10:**
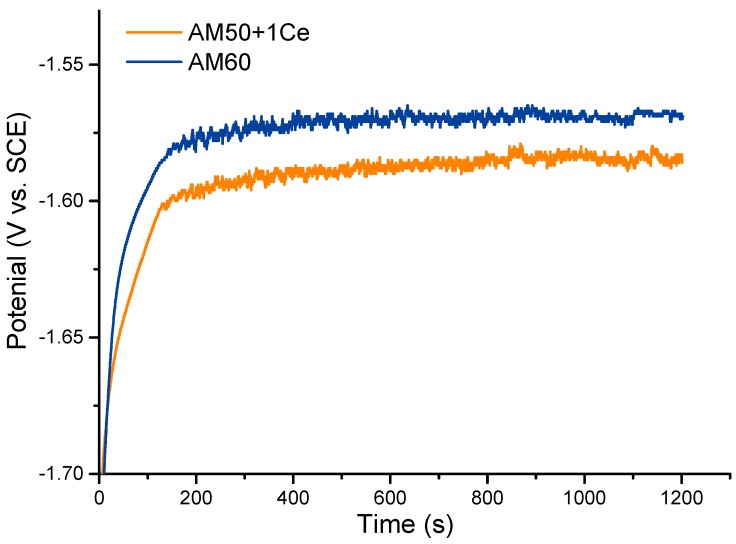
The open circuit potential of HPDC AM50+1Ce and AM60 magnesium in 3.5 wt% NaCl solution.

Figure 10 compares the open circuit potential (OCP) of HPDC AM50+1Ce and AM60 in 3.5 wt% NaCl solution. In the initial stage, the evolution of the OCP was closely related to the growth of the local corrosion of the alloy [34]. In the first 200 s, the OCP of the two in the 3.5 wt% NaCl aqueous solution was different, the surface of the working electrode was constantly polarized over time, the potential shifted towards positive direction and then the two reached the potential stable platform almost simultaneously. This was the result of dynamic balancing between the local corrosion and corrosion product. When AM50+1Ce reached steady state, the potential was around −1.585 vs. SCE, and the stable platform potential of AM60 was about −1.557 vs. SCE. The AM50+1Ce had more active electrochemical activity.

**Figure 11:**
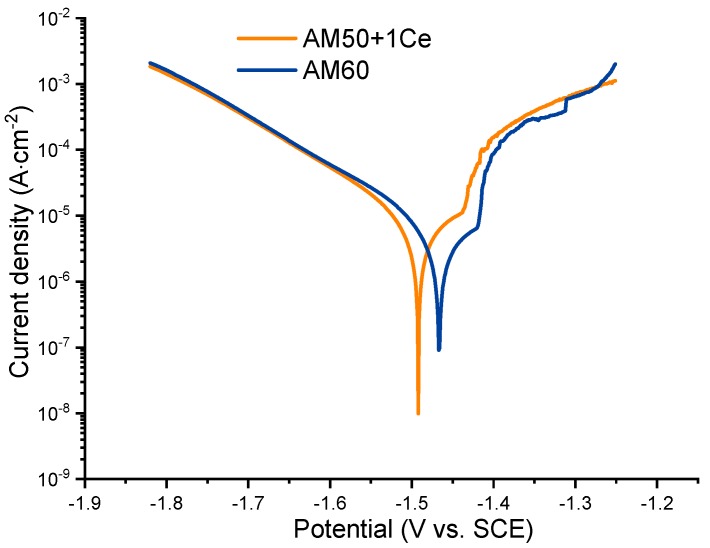
Potentiodynamic polarization behavior of HPDC AM50+1Ce and AM60 magnesium alloys in 3.5 wt% NaCl solution.

Figure 11 presents the polarization curves of HPDC AM50+1Ce and AM60 in 3.5 wt% NaCl solution. It was obvious that the cathode branch and the anode branch of the polarization curve were asymmetrical. In the process of anodic polarization, there was a small platform and a curve jump phenomenon, indicating the pitting and collapse of the passivation film, in the later stage, the AM60 anodic polarization branch fluctuated greatly, which relates to the pitting corrosion [23,35,36]. Moreover, in the anodic polarization process, the current density of AM50+1Ce was greater than that of AM60, indicating that the former was more severely corroded. Corresponding electrochemical parameters were calculated through the cathode branch of polarization curve by the Tafel extrapolation method, the corrosion current density (*i*_corr_) and corrosion potential (*E*_corr_) are summarized in Table 2. According to the fitting results, HPDC AM50+1Ce sample had a more negative corrosion potential (−1.522 V vs. SCE) and a larger corrosion current density (79.63 μA·cm^−2^), indicating a higher electrochemical activity. The corrosion potential (−1.497 V vs. SCE) of AM60 was more positive, and its corrosion current density (69.57 μA·cm^−2^) was also smaller, indicating that it had better corrosion resistance than the former one. This was consistent with the hydrogen evolution results. The corrosion rate of HPDC AM50+1Ce was larger than that of AM60.

**Figure 12:**
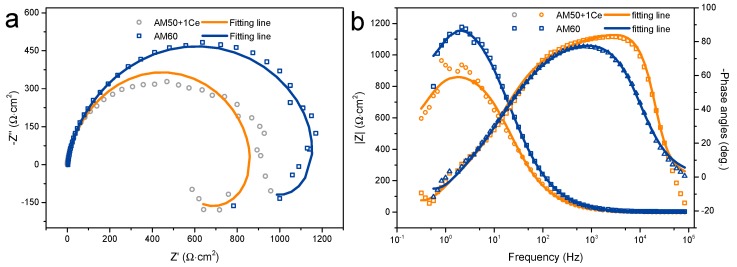
The Nyquist (**a**) and Bode plots (**b**) of EIS data of HPDC AM50+1Ce and AM60 magnesium alloys in 3.5 wt% NaCl solution.

**Figure 13:**
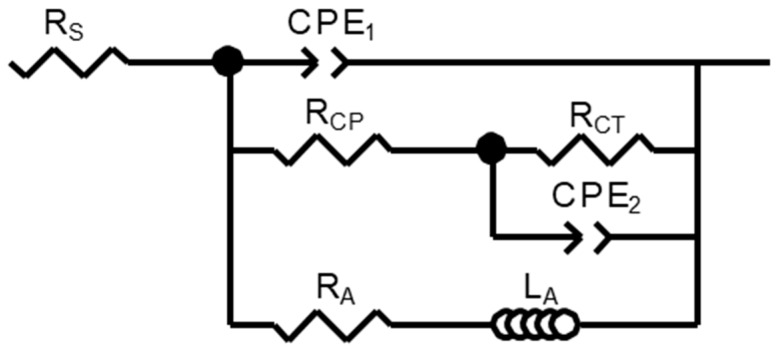
Equivalent electrical circuit used to fit the EIS spectra of HPDC AM50+1Ce and AM60 magnesium alloys.

The results of the EIS are set out in Figure 12. In the Nyquist plot, it is clear that AM60 had a larger capacitive arc than AM50+1Ce, indicating that AM60 had a better corrosion resistance than AM50+1Ce. Similar results could also be obtained from the Bode plot, in which the impedance modulus of AM60 was apparently higher than that of AM50+1Ce. The higher impedance modulus in the low frequency range indicates better corrosion resistance [8,9]. The impedance modulus (2 Hz) of AM60 and AM50+1Ce was about 1150 Ω⋅cm^2^ and 850 Ω⋅cm^2^, respectively. For both of them, the impedance modulus turned to decline after reaching the peak with a positive shift in phase angle in low frequency range, which is the character of inductive behavior. The EIS data was further analyzed by fitting the raw data using the equivalent circuit (Figure 13), Table 3 summarizes the value of all the element used to fit the EIS data. What stood out in this table was the difference between the two alloys, AM60 shows larger values in all resistance values. *R*_s_ is the solution resistance between the working electrode and the reference electrode [37], *R*_CT_ is the charge transfer resistance of the electrical double layer near the surface, which is a key parameter related to the corrosion performance of magnesium alloys. To fit the capacitive property of the interface, constant phase element (CPE) was introduced. CPE can both fit the capacitive property and enhance the fitting quality, which has been used widely in fitting EIS data [8]. *R*_CP_ represents the resistance characteristic of the corrosion products that cover part of the substrate surface, in the present study, the value of this element was quite small, which indicated the poor protective property of the passivation film. The *R*_CT_ combined with CPE_2_ represents the cathode reaction process of magnesium alloy, which was why the charge transferring in this process was a key parameter related to the corrosion. On the contrary, *R*_L_ and *L*_A_ represent the resistance and inductance related to the anode process of the corrosion [38]. Comparing the corrosion rate from weight loss, hydrogen evolution, corrosion current and EIS, we could see that the result was consistent excluding weight loss. As we mentioned above, the data of weight loss was close and easily influenced in the cleaning process, so according to the hydrogen evolution and electrochemical test, we came to the conclusion that the corrosion rate of AM50+1Ce was larger than AM60.

## 4. Discussion

**Figure 14:**
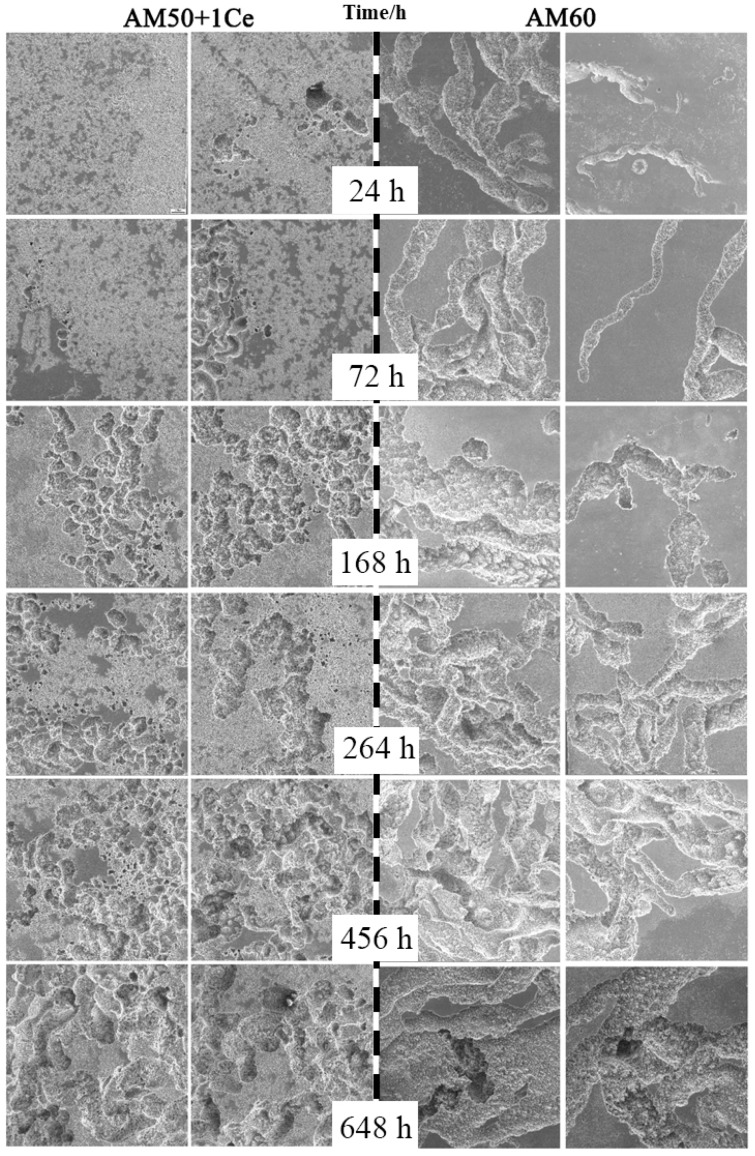
The corrosion morphology of HPDC AM50+1Ce and AM60 in 3.5 wt% NaCl solution for immersion of 24 h, 72 h, 168 h, 264 h, 456 h and 648 h.

The morphology and corrosion propagation of HPDC AM50+1Ce and AM60 after immersion for 24 h, 72 h, 168 h, 264 h, 456 h and 648 h in 3.5 wt% NaCl solution is displayed in Figure 14. Corrosion of both alloys began with pitting, but there was a noteworthy difference in corrosion propagation behavior. Local corrosion of AM60 was more obvious, and its macroscopic corrosion morphology was similar to the filamentous corrosion pit of AZ91. As the immersion time progressed, the corrosion progressed toward the two sides of the filament corrosion, the tip and the inside of the matrix, and the filamentous corrosion pits were connected to each other, continuously eroding the magnesium matrix. Local corrosion of HPDC AM50+1Ce was lighter, showing more corrosion nucleation points, more uniform corrosion spread and more uniform corrosion morphology, but there was still some local corrosion in the alloy. Moreover, as the immersion time prolonged, the etch holes continued to expand, the corrosion pits continued to grow, and the corrosion pits were connected to each other, thereby eroding the entire alloy surface, and finally forming corrosion appearance with different sizes, different depths, and uneven distribution. The main reason was that the solidification process of die-cast magnesium alloy is a non-equilibrium solidification process. There are components such as segregation and uneven structure in the alloy, which leads to different corrosion potentials in different parts. The protective film formation was different, and finally the difference of corrosion morphology presented.

**Figure 15:**
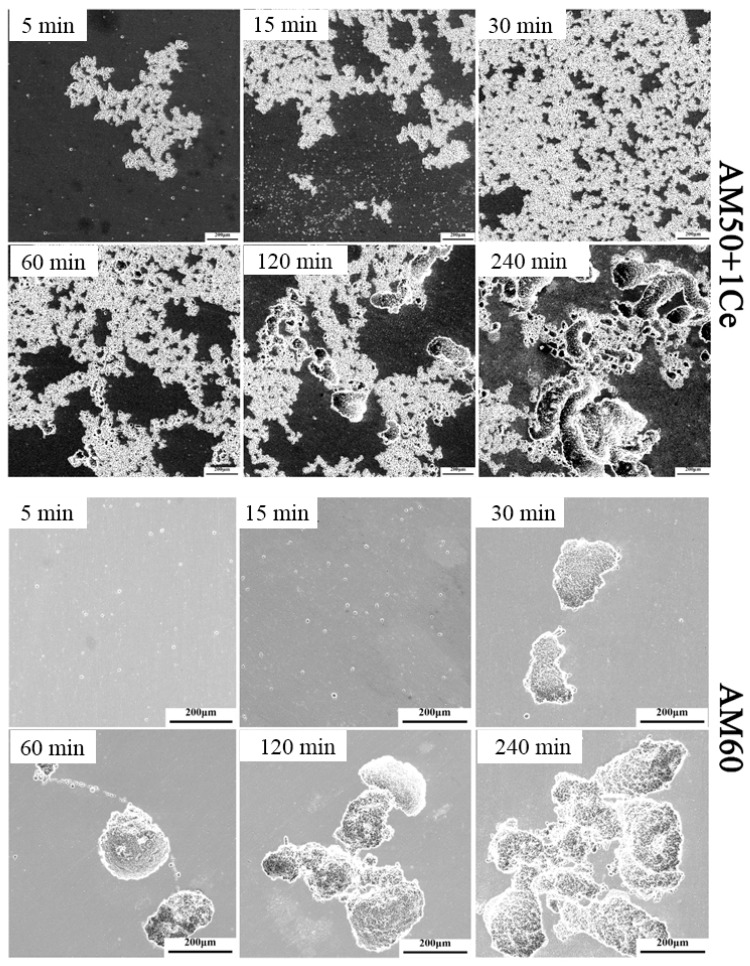
The corrosion morphology for HPDC AM50+1Ce and AM60 in immersion for different times.

To further investigate the corrosion behavior and expansion of two alloys, two sets of samples were immersed in the corrosion media for 5 min, 15 min, 30 min, 60 min, 120 min and 240 min, then the corrosion products were cleaned and observed. As shown in Figure 15, in the initial stage, AM50+1Ce corroded in a large area, the distance between each pitting was small, and the expansion speed of corrosion pit was faster. Corrosion pits quickly merged into larger corrosion pits, gradually eroded the surface layer of magnesium alloy, and the overall corrosion rate was faster, which was consistent with the previous hydrogen evolution results. The AM60 corroded very slowly in the initial stage, mainly concentrated in a small area, especially in casting defects such as shrinkage cavities and pores. It can be seen from Figure 15 that the pitting in the AM60 magnesium alloy continuously enlarged and deepened, the corrosion pits connected and merged, and the corrosion was still not observed in the area where no corrosion had occurred before, thus indicating local corrosion of AM60 was severe, which was consistent with previous electrochemical tests and corrosion residual strength results.

**Figure 16:**
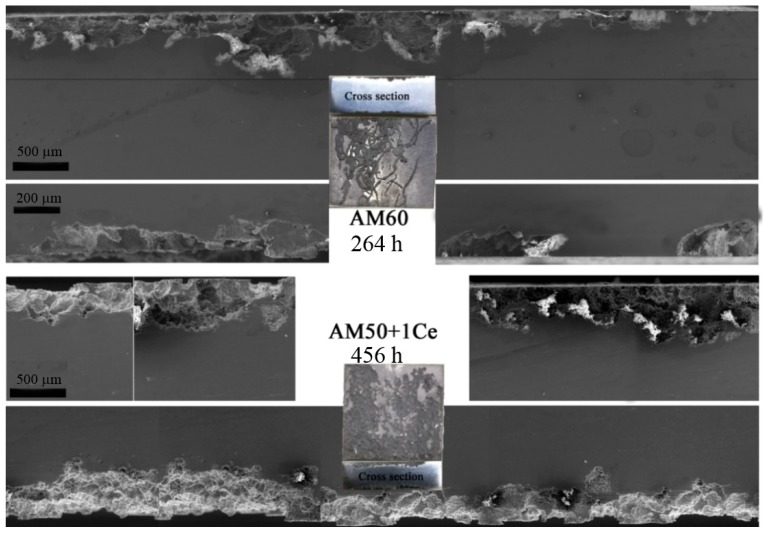
The cross-section corrosion morphology of HPDC AM50+1Ce and AM60 magnesium alloys immersed in 3.5 wt% NaCl solution for 456 and 264 h.

Figure 14 and Figure 15 show the surface corrosion characteristics of the AM50+1Ce and AM60, but the corrosion to the inside of the matrix was not clear. For this reason, weight loss samples of AM50+1Ce and AM60 after immersion time of 456 h and 264 h in 3.5 wt% NaCl solution were selected for cross-section observation, as presented in Figure 16. The results show that the corrosion of AM50+1Ce was more uniform to the whole of the matrix; while the local corrosion of AM60 was severe, indicating that the pitting effect was stronger, it can be observed that the corrosion pit was in the favorable direction during the expansion process. In particular, casting defects inside the alloy extend to form three-dimensional corrosion pits of various shapes. The area where the corrosion has not occurred was gradually eroded, and was removed during the cleaning process. At the same time, it can be observed that micro cracks were formed in the corroded areas, which was consistent with the observation in Figure 9, which would adversely affect the mechanical properties of the magnesium alloy.

**Figure 17:**
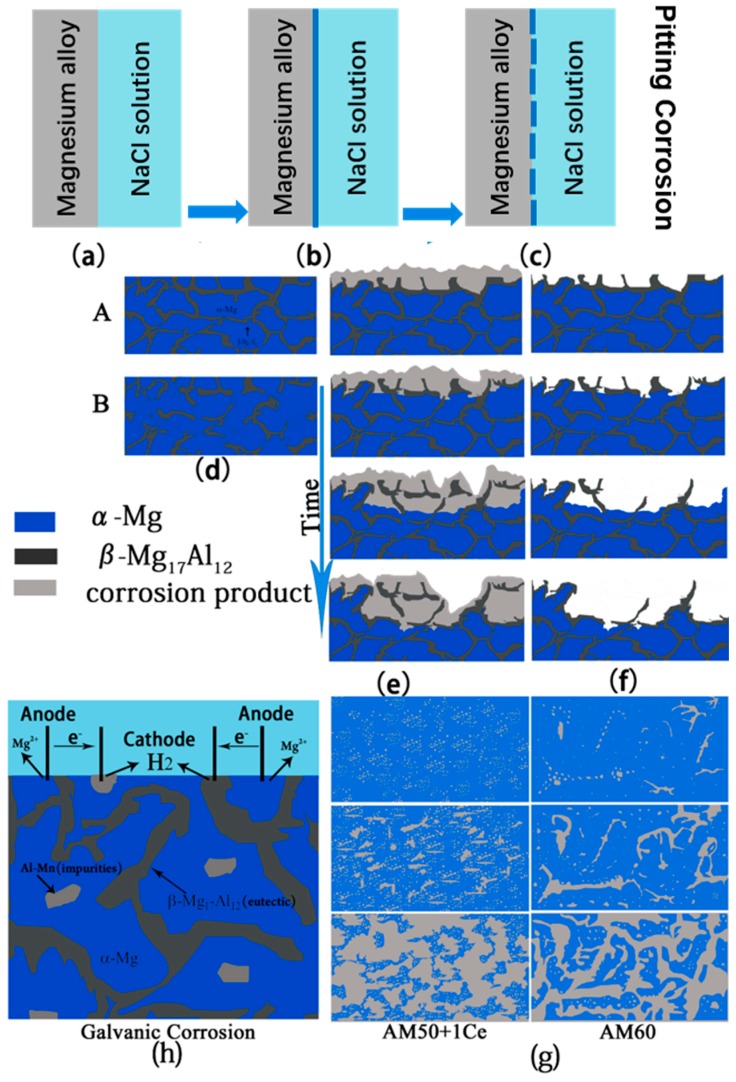
The schematic diagram of corrosion mechanism and morphology expansion model of HPDC AM50+1Ce and AM60 magnesium alloys in 3.5 wt% NaCl solution. (**a**–**c**) pitting process; (**d**) two type of second phase structure; the corrosion model before (**e**) and after (**f**) corrosion products cleaning; (**g**) corrosion propagation behavior of these two alloys and schematic diagram of galvanic corrosion.

Based on the analysis of corrosion behavior and corrosion morphology of HPDC AM50+1Ce and AM60, a corrosion mechanism and morphology extension model were established. As shown in Figure 17, a thin film formed when magnesium alloy came into contact with the corrosion solution. For HPDC Mg alloy, the defect such as gas pore can lead to the local corrosion and destruct the film, pitting corrosion occurs. With the dissolution of Mg, the concentration of Mg ions in the solution increases continuously. Due to the slow diffusion rate of Mg ions in the solution under static conditions, the concentration of Mg ion is very high near the surface of the sample, and the Mg easily reacts with OH^−^ to form Mg(OH)_2_, attaching to the surface of the magnesium alloy. However, this corrosion product layer is not dense enough, and Cl^−^ has the characteristics of small dimension, low hydration degree and fast moving speed, which makes it easy to pass through the Mg(OH)_2_ film. In addition, the film easily drops off from the surface, then the magnesium matrix is again exposed, and as the concentration of Cl^−^ increases, the damage is more serious, thereby aggravating the corrosion of the magnesium alloy.

As mentioned above, corrosion morphology of HPDC AM50+1Ce was relatively uniform, while the local corrosion of AM60 was more serious. Compositional unevenness caused by component segregation, the size of dendrites, the second phase and casting defects such as shrinkage cavities and pores all have different effects on the corrosion of die casting magnesium alloys [39]. For the characteristics of these two die casting magnesium alloys, the corrosion behavior can be summarized as follows: if a network of Mg_17_Al_12_ forms in a certain area of the alloy, it will become a barrier to repress corrosion, that is the situation A in Figure 17d; if no network Mg_17_Al_12_ forms, casting defect would play a key role, as shown in the situation B in Figure 17d. Corrosion tends to occur preferentially in casting defects such as shrinkage cavities and pores and in the second phase. As time goes on, the interior of the matrix was continuously eroded and spread in all directions, as shown in Figure 17e. After cleaning the corrosion products, the surface of the sample showed etched holes with different depth and size, as shown in Figure 17f. The second phase, the impurity particles, and even some of the oxidation products in the magnesium alloy could be the cathode, and the magnesium matrix acts as the anode to cause galvanic corrosion. As shown in Figure 17h, the phase of HPDC AM50+1Ce and AM60 magnesium alloys was mainly composed of α-Mg, Mg_17_Al_12_ and Al–Mn particles. Mg_17_Al_12_ and Al–Mn particles became cathodes, and α-Mg was the anode, thereby accelerating the corrosion of the magnesium alloy, and the second phase particles, impurities and even the magnesium matrix may have been peeled off from the surface of the matrix. The schematic diagram of the corrosion propagation behavior of these two alloys is presented in Figure 17g. Corrosion of AM50+1Ce and AM60 magnesium alloy in 3.5 wt% NaCl solution was mainly pitting corrosion, and for shrinkage and porosity, Mg_17_Al_12_ and Al–Mn particles are preferentially corroded. However, the microstructure and composition distribution of AM50+1Ce was more uniform than those of AM60, and the characteristics of less casting defects and fine pores made the pitting corrosion of all areas start at the same time. With the prolongation of corrosion time, these etched holes would continue to spread both around the surface layer and inside the matrix, and then join with the surrounding etch holes to merge into larger and deeper etch holes. In the process, there may have been falling off of the matrix. On the other hand, AM60 had coarse dendrites, the distribution of composition was more uneven, and the casting defects were much larger, it was because of this reason that its corrosion had more direction selectivity.

## 5. Conclusions

This study has examined the corrosion performance and corrosion residual strength of HPDC AM50+1Ce and AM60 Mg alloy. The corrosion model of HPDC AM50+1Ce and AM60 in 3.5 wt% NaCl solution has been preliminarily established, the following conclusions were obtained:

1. According to the hydrogen evolution and electrochemical tests, we conclude that the corrosion rate of AM50+1Ce was larger than AM60.
2. The elongation, yield strength and tensile strength of HPDC AM50+1Ce and AM60 declined with the increase of corrosion time. The residual strength of HPDC AM50+1Ce and AM60 after immersion for 648 h was 199 MPa and 183 MPa, respectively. The mechanical properties of HPDC AM60 were more sensitive to corrosion.
3. The corrosion of HPDC AM50+1Ce and AM60 in 3.5 wt% NaCl solution was mainly pitting corrosion. The corrosion of AM5+1Ce was more uniform, while AM60 had more serious local corrosion.

## Figures and Tables

**Table 1:** Actual composition of the tested alloys.

Designation	Al	Zn	Mn	Ce	Mg
AM50+1Ce	4.49	0.12	0.32	0.73	Bal.
AM60	5.71	0.15	0.27		Bal.

**Table 2:** Fitting results from polarization curves of HPDC AM50+1Ce and AM60 magnesium alloys in 3.5 wt% NaCl solution.

Alloy	*E*_corr_ (V vs. SCE)	*i*_corr_ (μA·cm^−2)^
AM50+1Ce	−1.522	79.63
AM60	−1.497	69.57

**Table 3:** Fitting results from polarization curves of HPDC AM50+1Ce and AM60 magnesium alloys in 3.5 wt% NaCl solution.

Circuit Elements	AM50+1Ce	AM60
*R*_s_ (Ω·cm^2^)	1.269	3.731
*R*_CT_ (Ω·cm^2^)	902.0	1246
CPE_2_ (s^n^/Ω·cm^2^)	2.161 × 10^−5^	2.112 × 10^−5^
*n*_2_	0.8603	0.7877
*R*_CP_ (Ω·cm^2^)	1.488	6.059
CPE_1_ (s^n^/Ω·cm^2^)	1.272 × 10^−8^	2.597 × 10^−7^
*n*_1_	1.555	1.252
*L*_A_ (H)	693.2	676.3
*R*_L_ (Ω·cm^2^)	1246	2705
